# A convenient detection system consisting of efficient Au@PtRu nanozymes and alcohol oxidase for highly sensitive alcohol biosensing†

**DOI:** 10.1039/d0na00002g

**Published:** 2020-02-27

**Authors:** Feng Lv, Yuzhu Gong, Yingying Cao, Yaoyao Deng, Shufeng Liang, Xin Tian, Hongwei Gu, Jun-Jie Yin

**Affiliations:** College of Chemistry, Chemical Engineering and Materials Science, Collaborative Innovation Center of Suzhou Nano Science and Technology, Collaborative Innovation Center for New-type Urbanization and Social Governance of Jiangsu Province, Soochow University Suzhou 215123 P. R. China hongwei@suda.edu.cn; School of Chemical Engineering and Materials, Changzhou Institute of Technology Changzhou 213032 P. R. China; State Key Laboratory of Radiation Medicine and Protection, School of Radiation Medicine and Protection, School for Radiological and Interdisciplinary Sciences (RAD-X), Collaborative Innovation Center of Radiation Medicine of Jiangsu Higher Education Institutions, Soochow University Suzhou 215123 China xtian@suda.edu.cn; Department of Molecular Biology, Shanxi Cancer Hospital and Institute, Affiliated Hospital of Shanxi Medical University Taiyuan Shanxi 030013 China; Division of Analytical Chemistry, Office of Regulatory Science, Center for Food Safety and Applied Nutrition, U.S. Food and Drug Administration College Park Maryland 20740 USA

## Abstract

Effective alcohol detection represents a substantial concern not only in the context of personal and automobile safety but also in clinical settings as alcohol is a contributing factor in a wide range of health complications including various types of liver cirrhoses, strokes, and cardiovascular diseases. Recently, many kinds of nanomaterials with enzyme-like properties have been widely used as biosensors. Herein, we have developed a convenient detection method that combines Au@PtRu nanozymes and alcohol oxidase (AOx). We found that the Au@PtRu nanorods exhibited peroxidase-like catalytic activity that was much higher than the catalytic activities of the Au and Au@Pt nanorods. The Au@PtRu nanorod-catalyzed generation of hydroxyl radicals in the presence of H_2_O_2_ was used to develop an alcohol sensor by monitoring the H_2_O_2_ formed by the oxidation of alcohol to acetaldehyde in the presence of AOx. When coupled with AOx, alcohol was detected down to 23.8 μM in a buffer solution for biological assays. Notably, alcohol was successfully detected in mouse blood samples with results comparable to that from commercial alcohol meters. These results highlight the potential of the Au@PtRu nanorods with peroxidase-like activity for alcohol detection, which opens up a new avenue for nanozyme development for biomedical applications.

## Abstract 2

A convenient alcohol detection method involving efficient Au@PtRu nanozymes and alcohol oxidase was developed.

## Graphical abstract

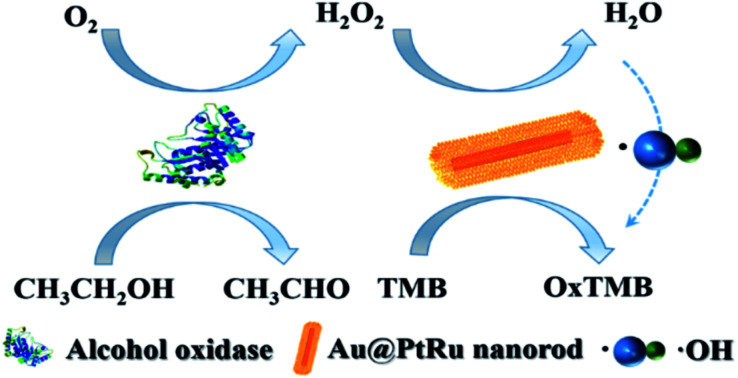

## Introduction

Alcohol, a common medicine and drink, can cause a variety of hypersensitive reactions, health complications, and drunkenness, which are always endangering transportation safety.^1–3^ Therefore, there is great interest in alcohol biosensing not only in personal and automobile safety, but also in pharmaceutical and clinical settings.^4–6^ Currently, highly sensitive and reliable alcohol detection is typically available through precision instrument-based analyses confined mostly to laboratory facilities. For example, the commonly used alcohol analytical methods include gas chromatography,^7^ high-performance liquid chromatography^8^ and redox titrations.^9^ In addition, portable sensors of alcohol have been widely used for the rapid detection of alcohol for automobile safety and clinical assays.^10^ Among these methods, ethanol conversion enzyme-based alcohol detection methods are widely developed.^11,12^

Recently, many kinds of nanomaterials (also called nanozymes) with enzyme-like properties have been widely used as biosensors, including the nanomaterials of carbon,^13,14^ metals,^15–17^ and transition metal oxides.^18–21^ Among them, noble metal nanomaterials, such as gold (Au), platinum (Pt), and ruthenium (Ru), have attracted more attention because of their high catalytic activities, tunable morphology, and excellent biocompatibility.^11,22,23^ For example, BSA-stabilized Au clusters have been developed as sensitive biosensors for xanthine detection based on the peroxidase-like activity of the Au clusters.^24^ In addition, Wang and co-workers have successfully detected biothiols and proteins and discriminated cancer cells by using peroxidase-like Pt and Ru nanozyme-constructed sensor arrays.^25^

However, the detection sensitivity of noble metal nanomaterials still needs to be improved; further enhancing the catalytic efficiency of nanoparticles is crucial for developing potential biosensors. Compared with single-component noble metal nanomaterials, trimetallic noble metal nanomaterials can provide a higher chance for enhancement in catalytic efficiency.^26–28^ In this study, we detected alcohol using Au@PtRu nanorods. These Au@PtRu nanorods possess highly efficient peroxidase-like activity that is much higher than that of the Au and Au@Pt nanorods. When the Au@PtRu nanorods were coupled with alcohol oxidase (AOx), we achieved the ultrasensitive detection of alcohol in a buffer solution and mouse blood. To the best of our knowledge, there are no prior reports of the colorimetric detection of alcohol based on trimetallic core–shell structured nanoparticles.

## Experimental

### Chemicals and materials

Hexachloroplatinic acid hexahydrate (H_2_PtCl_6_·6H_2_O), potassium hexachloroplatinate (K_2_PtCl_4_), silver nitrate (AgNO_3_), chloroauric acid tetrahydrate (HAuCl_4_·4H_2_O), ruthenium trichloride (RuCl_3_), hexadecyl trimethylammonium bromide (CTAB), methanol (CH_3_OH), ethanol (CH_3_CH_2_OH), 1-propanol (CH_2_OHCH_2_CH_3_), 2-propanol (CH_3_CHOHCH_3_), 1-butanol (CH_2_OHCH_2_CH_2_CH_3_), and hydrogen peroxide (H_2_O_2_, 30%) were purchased from Sinopharm Chemical Reagent Co. Ltd., China. 3,3′,5,5′-Tetramethylbenzidine (TMB) and dimethyl sulfoxide (DMSO) were purchased from Aladdin Bio-Chem Technology Co. Ltd., China. Alcohol oxidase (40 U per mg protein) was purchased from Shanghai yuanye Bio-Technology Co. Ltd., China. Pluronic F127 (PEO_100_PPO_65_PEO_100_) was obtained from Macklin Biochemical Co. Ltd., China. Sodium borohydride (NaBH_4_), *o*-phenylenediamine (OPD) and 2,2′-azinobis(3-ethylbenzthiazoline-6-sulphonate) (ABTS) were purchased from Alfa Aesar (China).

### Synthesis of Au nanorods

Au nanorods were prepared *via* a seed growth method. First, the Au seeds were prepared by adding 25 μL HAuCl_4_ (10 mM) to a 7.5 mL CTAB (100 mM) aqueous solution using NaBH_4_ as the reducing agent. The seed solution was kept at room temperature for 30 min prior to any further experiment. Then, the growth solution of the Au nanorods was prepared by adding 2.04 mL HAuCl_4_ (10 mM), 40 μL HCl (12 M), 200 μL AgNO_3_ (10 mM), and 800 μL ascorbic acid (100 mM) to 45 mL of CTAB (100 mM) aqueous solution and stirring the solution until it became colorless. Finally, 420 μL of the seed solution was added into the growth solution and reacted overnight. The Au nanorods were collected by centrifugation at 10 000 rpm for 20 min.

### Synthesis of Au@Pt and Au@PtRu nanorods

1 mL of the above-mentioned Au nanorod suspension was mixed with 720 μL K_2_PtCl_4_ (20 mM), 1.08 mL H_2_PtCl_4_ (20 mM), 600 μL RuCl_3_ (20 mM), 30 μL HCl (12 M) and 60 mg F127. The mixture solution was sonicated until it became homogeneous. Then, 5 mL ascorbic acid (100 mM) was added into the abovementioned solution and sonicated for 2 h at 80 °C. The products (Au@PtRu) were washed three times with deionized water. To obtain Au@Pt nanoparticles, RuCl_3_ was removed from the reaction. The Au@PtRu and Au@Pt nanorods were collected by centrifugation at 8000 rpm for 5 min.

### Peroxidase-like activity

For the oxidation of TMB, the reactions were carried out in 750 μL NaAc–HAc buffer solution (200 mM, pH 3.6), 50 μL nanozymes (0–50 μg mL^−1^), 100 μL TMB (40 mM) and 100 μL H_2_O_2_ (1 M). The absorption spectra of the samples were recorded using a UV spectrometer.

The specific activity (SA) is defined as activity units per milligram of a nanozyme, which can be obtained using the following equation:

1 SA = *V*/(*ε* × *l*) × (Δ*A*/Δ*t*) × *m*

Here, *V* is the total volume of the reaction solution (μL); *ε* is the molar absorption coefficient of TMB, whose value is 39 000 M^−1^ cm^−1^; and *m* is the nanozyme weight (mg) of each assay.

### Kinetic measurements of TMB oxidation

We placed 2 mL of a solution containing 5 μg mL^−1^ nanozymes and 100 mM H_2_O_2_ in NaAc–HAc buffer (200 mM, pH 3.6). TMB was introduced into the reaction system. We then mixed the samples and recorded the initial rate of the change in absorbance at 652 nm. The kinetic constants were calculated based on the Michaelis–Menten equation as follows:

2 *V* = *V*_max_ × [S]/(*K*_m_ + [S])

*V*: initial reaction velocity; *V*_max_: maximal reaction rate; [S]: substrate concentration; and *K*_m_: Michaelis constant.

### The detection of hydroxyl radicals

Terephthalic acid (TA) was used as a fluorescent probe to detect the generation of hydroxyl radicals (˙OH). Ten μL TA (5 mM) was mixed with 790 μL NaAc–HAc buffer solution (pH 3.6), 100 μL nanozymes, and 100 μL H_2_O_2_ (1 M). The mixed solution was allowed to react for 6 h and then, the fluorescence spectra of the samples were collected using fluorescence spectroscopy.

### Alcohol oxidase-based alcohol sensing

First, 50 μL alcohol samples (methanol, ethanol, 1-propanol, 1-butanol, and 2-propanol) were mixed with 50 μL alcohol oxidase (AOx, 50 U mL^−1^) for 30 min. Then, 750 μL NaAc–HAc buffer (pH 3.6), 50 μL TMB (40 mM), and 50 μL Au@PtRu nanorods (20 μg mL^−1^) were added into the abovementioned solution. After incubating for another 30 min, the absorbance of each sample was recorded at 652 nm. The limit of detection (LOD) was computed from the standard deviation of the sensor response (*S*_y_) and the slope (*m*). We conducted 9 parallel trials of the sensors (UV-Vis spectrometer) to calculate the relative mean deviation to get Sy. The LOD was defined as 3.3 × *S*_y_/*m*. Mouse blood was taken from an adult C57BL/6 mouse and stored in anticoagulant tubes. Mouse blood was first attenuated 10 times by phosphate buffer saline (10 mM, pH 7.4). The standard concentration gradients of ethanol were prepared by mouse blood. As the mouse blood was introduced into the sensing system, the detection was followed using the same procedure as above.

### Apparatus

The surface morphology and composition characterization were achieved using a transmission electron microscope (TEM) (Tecnai G20, FEI, USA). The high-resolution TEM (HRTEM), high-angle annular dark-field scanning TEM (HAADF-STEM), elemental mapping and energy dispersive X-ray (EDX) analyses of the samples were performed by a Tecani F20 instrument (FEI, USA). The X-ray diffraction (XRD) patterns were obtained using an X'Pert-Pro MP diffractometer (Malvern PANalytical, Netherlands) and the precise elemental content was tested by an iCAP6300 instrument (Thermo Fisher Scientific, USA). The parameters of peroxidase-like activity were achieved by a UV-Vis spectrometer (UV-3600, Shimadzu, Japan). The fluorescence spectra of the TA tests were obtained using fluorescence spectroscopy (FLS980, Edinburgh, UK). X-ray photoelectron spectroscopy (XPS) was performed by an electron energy analyzer (Escalab250Xi, UK). The device used for nanorod sonication was an ultrasonic cleaner (SK7200H, 100 Hz, Kunshan ultrasonic Instruments China).

## Results and discussion

**Fig. 1:**
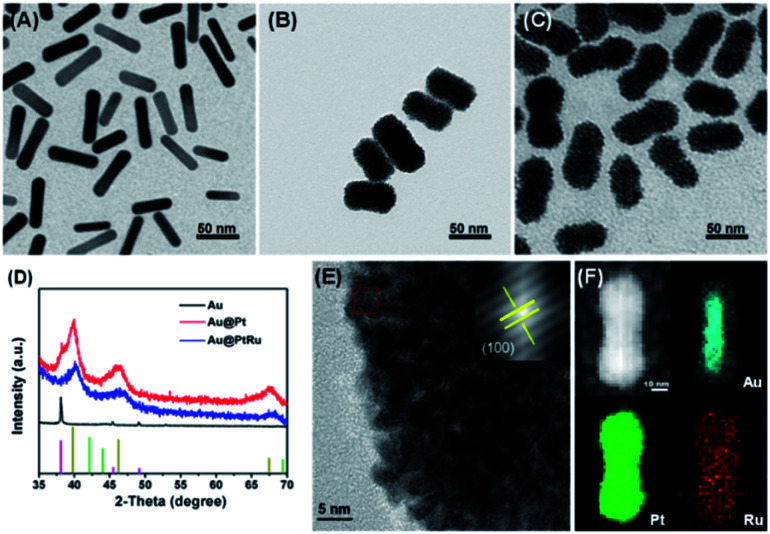
Typical TEM images of Au (A), Au@Pt (B), and Au@PtRu nanorods (C). (D) XRD patterns of Au, Au@Pt, and Au@PtRu nanorods. (E) HRTEM image and the corresponding SAED pattern (inset) of Au@PtRu nanorods. (F) HAADF-STEM images and EDX elemental mapping images of Au@PtRu nanorods.

The Au nanorods were synthesized using a seed growth method^29,30^ and then employed as templates to guide the synthesis of Au@Pt and Au@PtRu nanorods. As shown in Fig. 1A–C, the Au, Au@Pt and Au@PtRu nanorods have uniform morphology with the aspect ratios of approximately 4.0 (length: 33.6 nm, width: 8.3 nm), 2.2 (length: 58 nm, width: 26 nm), and 2.3 (length: 65 nm, width: 28 nm), respectively. The Au@Pt and Au@PtRu nanorods displayed silkworm cocoon-like structures with obvious micropores.

XRD patterns further supported the formation of the Au@Pt and Au@PtRu hybrid structures (Fig. 1D). The diffraction pattern of the Au@Pt nanorods had three peaks at 39.8°, 46.2° and 67.5°, corresponding to the (111), (200) and (220) planes of *syn* Pt. The diffraction pattern for the Au@PtRu nanorods also had three broad peaks, which fell between the corresponding diffraction peaks of pure Pt and Ru. These signals demonstrated the formation of the PtRu alloys. It is worth noting that a small peak appeared at 38.2°, corresponding to the (111) plane of *syn* Au, which further confirmed the specific structure of the Au@PtRu nanorods. The XPS results demonstrated that most Pt species in the Au@PtRu nanorods existed in the form of zero valent Pt and a small amount of Pt was in the form of Pt^2+^ (Fig. S1†).

The HRTEM results and selected area electron diffraction (SAED) patterns of the Au@Pt nanorods unveiled clear lattice fringes, corresponding to the (200) plane of the face-centered cubic (FCC) Pt alloy (Fig. 1E and S2†). Additionally, the lattice spacing of the Au@PtRu nanorods was 0.2069 nm, which was between the (200) interlayer spacing of Pt and the (100) interlayer spacing of Ru. This result could be attributed to an alloy of Pt and Ru.^31^ We further analyzed the Au@Pt and Au@PtRu nanorods with STEM coupled with EDX (Fig. 1F and S3†). An overlay of the Pt and Ru EDX maps is shown in Fig. 1F, indicating a clear homogenous distribution of the two elements over the Au nanorods and confirming the successful formation of the core–shell structure of the Au@PtRu nanorods. The compositional line profiles of a single Au@PtRu nanorod are shown in Fig. S4,† which clearly present the distribution of Au, Pt, and Ru. Inductively coupled plasma atomic emission spectroscopy (ICP-AES) was used to determine the molar ratios of Au, Pt and Ru in the Au@Pt and Au@PtRu nanorods, which were Au_8_Pt_92_ and Au_7_Pt_74_Ru_19_, respectively.

The growth process of the Au@PtRu nanorods was monitored by TEM (Fig. S5†). In the first 30 min, small particles adhered to the surface of the Au nanorods. As the time increased to 120 min, an increasing number of these particles gradually accumulated on the Au nanorods, which finally resulted in a porous silkworm cocoon-like structure. During the reaction, the micelles formed by F127, which contained the precursor solution of Pt and Ru, were reduced by AA, leading to the formation of these small PtRu particles.^32^ These PtRu particles consistently deposited on the Au rods and finally led to the formation of PtRu alloying layers over the Au rods.

**Fig. 2:**
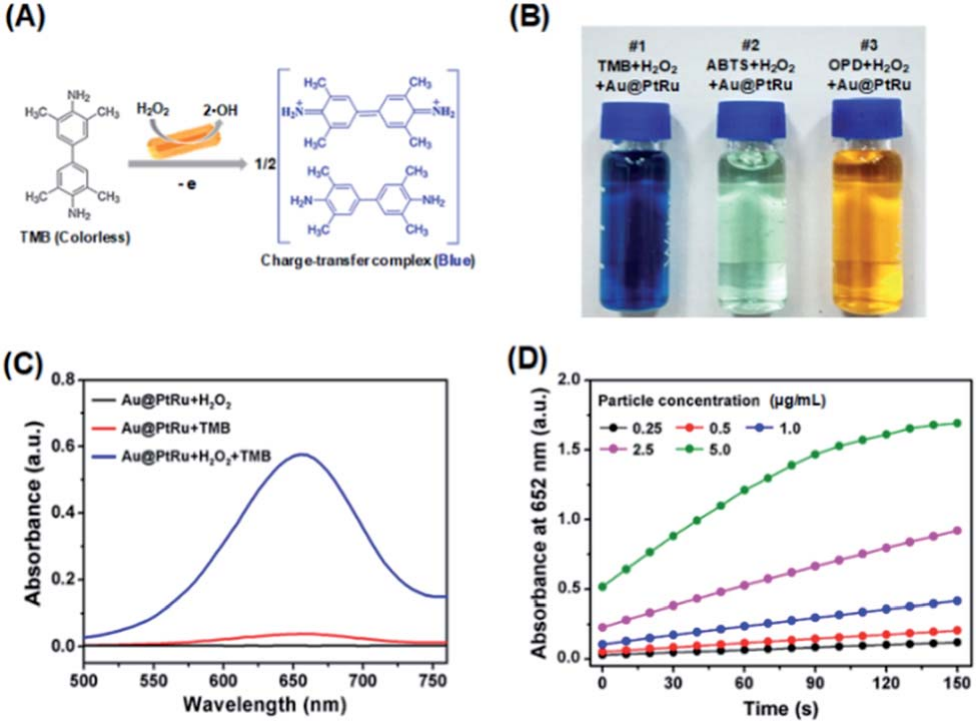
Peroxidase-like activity of the Au@PtRu nanorods. (A) Scheme showing the oxidation of TMB by the Au@PtRu nanorods in the presence of H_2_O_2_. (B) A photograph showing the capability of the Au@PtRu nanorods to catalyze the oxidation of various peroxidase substrates (*i.e.*, TMB, ABTS, and OPD). (C) UV-Vis spectra for the detection of peroxidase-like activity of the Au@PtRu nanorods (5 μg mL^−1^) from the different reaction systems. Au@PtRu nanorods: 5 μg mL^−1^, TMB: 4 mM, H_2_O_2_: 100 mM, NaAc–HAc buffer solution: 200 mM, pH 3.6. (D) Absorbance at 652 nm measured from the reaction solutions containing TMB (4 mM), H_2_O_2_ (100 mM), and Au@PtRu nanorods with different concentrations in the NaAc–HAc buffer solution (200 mM, pH 3.6).

**Fig. 3:**
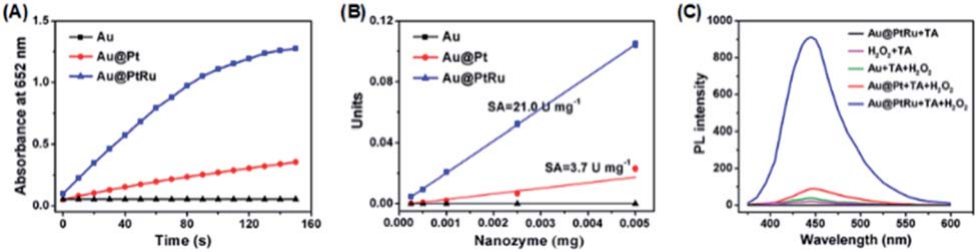
(A) Time-dependent absorbance at 652 nm measured from the reaction solutions containing TMB, H_2_O_2_, and Au nanorods, Au@Pt nanorods, or Au@PtRu nanorods. (B) The specific activities of the Au, Au@Pt, and Au@PtRu nanorods. (C) Fluorescence spectra for the detection of ˙OH from the different reaction systems.

Then, the peroxidase-like activity of the Au@PtRu nanorods was determined. A commonly used peroxidase substrate is TMB. H_2_O_2_ can be catalyzed by peroxidase to generate hydroxyl radicals (˙OH), which then oxidize TMB to a blue-colored product (maximum absorbance: 652 nm) (Fig. 2A). As shown in Fig. 2B and C, the Au@PtRu nanorods catalyze the oxidation of TMB in the presence of H_2_O_2_, yielding a blue-colored product. The reaction rate was directly proportional to the particle concentration of the Au@PtRu nanorods (Fig. 2D). In addition to TMB, the Au@PtRu nanorods catalyzed the oxidation of several other peroxidase substrates such as ABTS and OPD, producing green- and orange-colored products, respectively (Fig. 2B). We found that the catalytic efficiency of the Au@PtRu nanorods for TMB depended on time, pH and temperature (Fig. S6 and S7†). As displayed in Fig. 3A, the Au@PtRu nanorods show good peroxidase-like activity for the oxidation of TMB, as evidenced by the time dependence of the maximum absorbance (652 nm). Consistently, the Au@Pt nanorods displayed an oxidation profile similar to that of the Au@PtRu nanorods but exhibited much weaker capacity to oxidize TMB. In contrast, the Au nanorods showed no ability to oxidize TMB, which was consistent with a previous report that the Au nanorods have no peroxidase-like activity.^33,34^ The specific activity (SA), which is defined as activity units (U) per milligram of a nanozyme, is an important standard for evaluating the peroxidase-like catalytic activity of the nanozyme.^35^ Therefore, we determined the SA values of the Au, Au@Pt, and Au@PtRu nanorods. As can be seen from Fig. 3B, the SA values of the Au@PtRu nanorods are approximately 6-fold higher than those observed for the Au@Pt nanorods, suggesting that the Au@PtRu nanorods have higher peroxidase-like activity. Nanozymes with peroxidase-like activity can generate ˙OH in the presence of H_2_O_2_. To verify the peroxidase-like property of the Au@PtRu nanorods, we followed the intermediates of the reaction, specifically ˙OH.^36–38^ The formation of ˙OH was monitored with terephthalic acid (TA), a typical fluorescent probe for the detection of ˙OH. In the presence of H_2_O_2_, the Au@Pt and Au@PtRu nanorods catalyzed the fluorescence of TAOH, indicating the formation of ˙OH (Fig. 3C). The Au@PtRu nanorods enhanced the generation of ˙OH more than the Au@Pt nanorods. Under these conditions, the Au nanorods produced little to no ˙OH. These findings suggest the highly efficient peroxidase-like activity of the Au@PtRu nanorods.

**Fig. 4:**
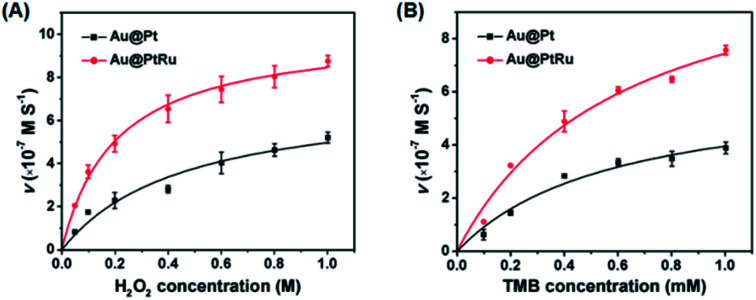
Kinetic analysis for the Au@Pt and Au@PtRu nanorods with H_2_O_2_ (A) and TMB (B) as substrates, respectively.

**Fig. 5:**
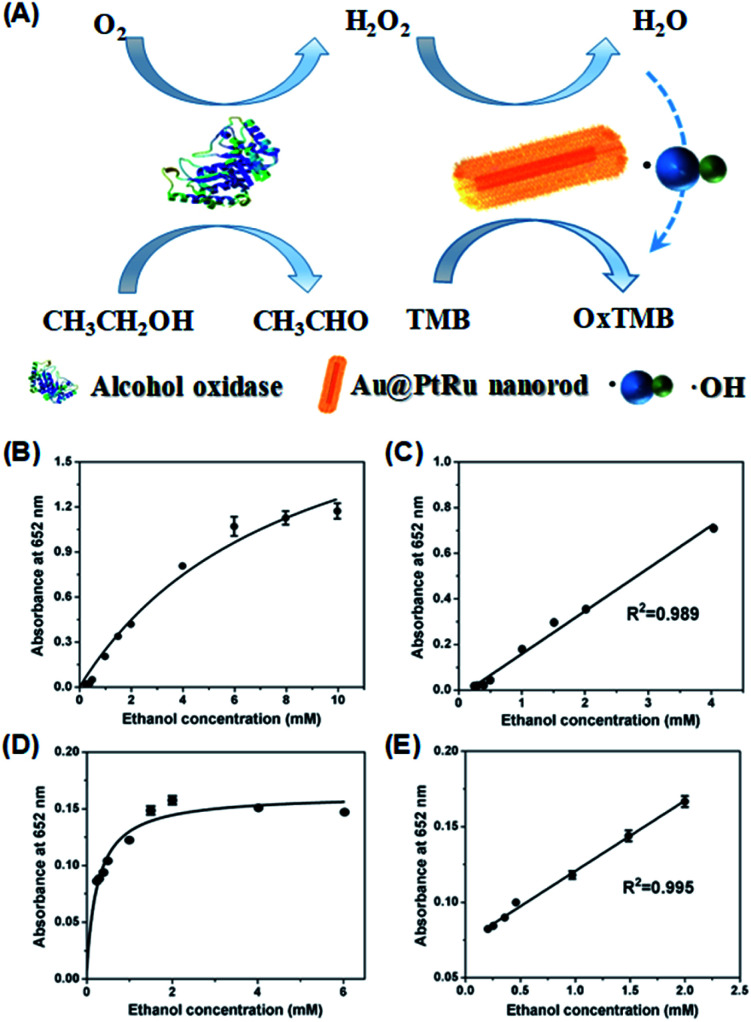
50 μL ethanol standards were incubated with 50 μL AOx (50 U mL^−1^) for 30 min. Then, 750 μL NaAc–HAc buffer (200 mM, pH 3.6), 50 μL TMB (40 mM), and 50 μL Au@PtRu nanorods (20 μg mL^−1^) were added into the abovementioned solution. After incubating for another 30 min, absorbance of each sample was recorded at 652 nm. (A) Schematic illustration of the catalytic detection of alcohol by Au@PtRu nanorods. Calibration curve and linear calibration curve for ethanol detection in the NaAc–HAc buffer solution (B and C) and mouse blood samples (D and E).

**Fig. 6:**
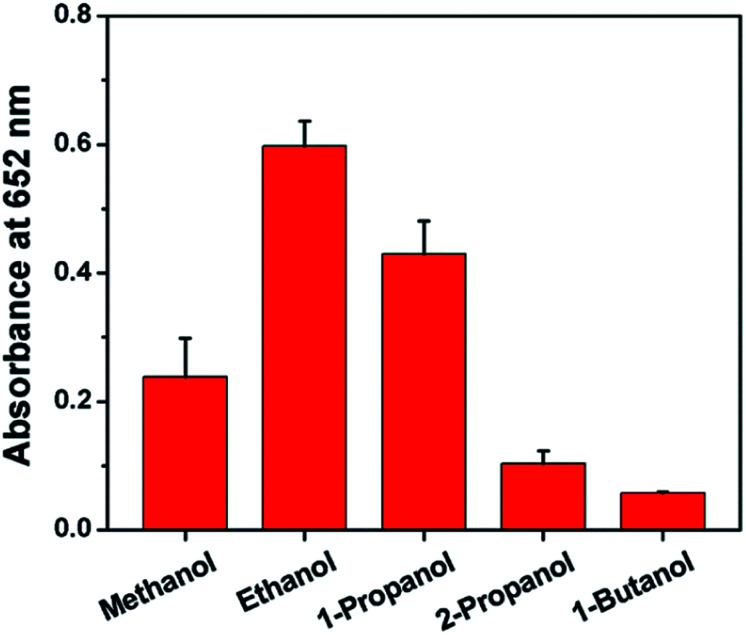
Alcohol samples were mixed with AOx (50 U mL^−1^). Then, NaAc–HAc buffer (200 mM, pH 3.6), TMB (40 mM), and Au@PtRu nanorods (20 μg mL^−1^) were added into above solution. After incubating for 30 min, absorbance of each sample was recorded at 652 nm.

To further quantify the catalytic efficiency, the apparent steady-state kinetic parameters of the Au@PtRu nanozyme were measured. Typical Michaelis–Menten curves were obtained in a certain range of TMB and H_2_O_2_ concentrations. With the Lineweaver–Burk equation, we calculated the enzyme kinetic parameters, including the Michaelis–Menten constant (*K*_m_) and the maximal reaction velocity (*V*_max_).^39,40^ As shown in Fig. 4 and Table 1, the apparent *K*_m_ (further *K*_m_) value of the Au@PtRu nanorods with H_2_O_2_ as the substrate is significantly lower than that of the Au@Pt nanorods, suggesting that the Au@PtRu nanorods have higher affinity for H_2_O_2_. Additionally, the catalytic efficiency (*k*_cat_/*K*_m_) of the H_2_O_2_ decomposition catalyzed by the Au@PtRu nanorods was more than 5-fold that of the Au@Pt nanorods, indicating that the Au@PtRu nanorods have excellent catalytic efficiency. Compared to most of the reported nanozymes, the Au@PtRu nanorods show higher peroxidase-like activity (Table S1†). The previously reported nanozymes were mainly composed of metal ions, and their peroxidase activities were owing to the variations in the valence of metal ions.^41^ The exposed metal valence of Pt and Ru is zero. Therefore, the peroxidase catalytic mechanism of the Au@Pt and Au@PtRu nanorods was different from that of the aforementioned nanozymes and more like a kind of electrochemical reduction. The free electrons combined with H_2_O_2_ to produce ˙OH, which then oxidized TMB. In addition, the porous structure of the Au@PtRu nanorods provided numerous catalytic active sites.^42^ The heterogeneous nucleation and the subsequent steady growth over the Au nanorods promised a stabilized structure of the Au@PtRu nanorods; meanwhile, they exerted good catalytic properties during the reaction.^43^ To demonstrate the applications of the Au@PtRu nanorods, the performance of an Au@PtRu nanorod-based biosensor to detect alcohol was studied under the optimized conditions. Alcohol was oxidized to acetaldehyde and H_2_O_2_ in the presence of AOx (Fig. 5A). The H_2_O_2_, which was generated by the AOx and ethanol, was then oxidized by the Au@PtRu nanorods and subsequently oxidized TMB to a blue-colored product. To confirm that ethanol was catalyzed by AOx instead of the Au@PtRu nanorods, the experiment was carried out without AOx (Fig. S8†). We found that the absorbance at 652 nm had no obvious change within 10 min. This result suggested that the Au@PtRu nanorods had no alcohol oxidase-like activity in our experimental condition. As shown in Fig. 5B, the responses of the Au@PtRu nanorod-based biosensor to ethanol concentrations ranging from 0.25 to 10 mM are assayed. It is worth noting that the change in OD at 652 nm exhibited a perfect linear relationship with ethanol concentrations ranging from 0.25 to 4 mM and the limit of detection (LOD) was 23.8 μM (Fig. 5C). Furthermore, the Au@PtRu nanorod-based biosensor was applied to detect alcohol in mouse blood samples. The sensor response for ethanol concentrations ranging from 0.25 mM to 6 mM was linear over the range of 0.25–2.0 mM with the LOD of 0.11 mM (Fig. 5D and E). Compared with the detection methods in other reports, our method shows a wider linear range and higher sensitivity in a pure solution.^44–47^ In addition, our results showed that the detection of alcohol was successfully carried out in mouse blood with results (LOD = 0.11 mM) comparable to that from commercial alcohol meters.^48,49^ Therefore, the Au@PtRu nanorod-based biosensor had adequate sensitivity for the detection of alcohol under physiological conditions. The detection of alcohols with varying lengths and branching (methanol, ethanol, 1-propanol, 2-propanol and 1-butanol) by the Au@PtRu nanorod-based biosensor was further tested. Fig. 6 shows that the biosensor responds differently to alcohol samples and exhibits the highest response when the substrate is ethanol. This further validates the potential of the Au@PtRu nanorod-based biosensor for the detection of biologically relevant metabolites.

**Table 1:** Comparison of the kinetic parameters of Au@Pt and Au@PtRu nanorods for the oxidation of TMB by H_2_O_2_^a^

Catalyst	[E] μM	Substrate	*V*_max_ (M s^−1^)	*K*_m_ (mM)	*k*_cat_ (s^−1^)	*k*_cat_/*K*_m_ (s^−1^ mM^−1^)
Au@PtRu	32.5 × 10^−6^	TMB	1.3 × 10^−6^	0.7	3.4 × 10^5^	4.9 × 10^5^
		H_2_O_2_	1.5 × 10^−6^	0.23 × 10^3^	4.0 × 10^5^	17.4 × 10^2^
Au@Pt	32.5 × 10^−6^	TMB	5.5 × 10^−7^	0.45	1.5 × 10^5^	3.3 × 10^5^
		H_2_O_2_	1.4 × 10^−6^	1.2 × 10^3^	3.7 × 10^5^	3.1 × 10^2^

a [E] is the concentration of nanozyme. The particle number of Au@PtRu or Au@Pt nanozyme is calculated using the density and diameter of the Au-rod core. Dividing the particle number by the Avogadro constant gives the molar concentration of the nanozyme. *K*_m_: Michaelis constant; *V*_max_: maximal reaction velocity; *k*_cat_: catalytic constant; *k*_cat_/*K*_m_: catalytic efficiency.

## Conclusions

In conclusion, we have developed a convenient alcohol detection method, which takes the advantage of the peroxidase-like activity of the Au@PtRu nanorods. When coupled with AOx, it is possible to detect alcohol even in mouse blood samples. This study opens up many new ways of using H_2_O_2_ for interfacing with bimetallic or trimetallic core–shell structured noble metal nanomaterials and also expands the scope of nanozyme biosensors.

## Ethics statement

All animal procedures were performed in accordance with the Guidelines for Care and Use of Laboratory Animals of Soochow University and experiments were approved by the Animal Ethics Committee of Soochow University.

## Conflicts of interest

There are no conflicts to declare.

## Supplementary Material

NA-002-D0NA00002G-s001
