# Supplementary material for: A convenient detection system consisting of efficient Au@PtRu nanozymes and alcohol oxidase for highly sensitive alcohol biosensing
Source: Nanoscale Adv. 2020 Feb 27;2(4):1583–9. doi: 10.1039/d0na00002g (PMC9416945; doi:10.1039/d0na00002g)
Supplement: NA-002-D0NA00002G-s001 [file NA-002-D0NA00002G-s001.pdf]

This journal is © The Royal Society of Chemistry 2020

Supporting Information

A Convenient Detection System Consisting of Efficient Au@PtRu Nanozymes and Alcohol Oxidase for Highly Sensitive Alcohol Biosensing

Feng Lv[a], Yuzhu Gong[a], Yingying Cao[a], Yaoyao Deng[b], Shufeng Liang[c,d,] Xin Tian[c,]*, Hongwei Gu[a,]*, and Jun-Jie Yin[e]

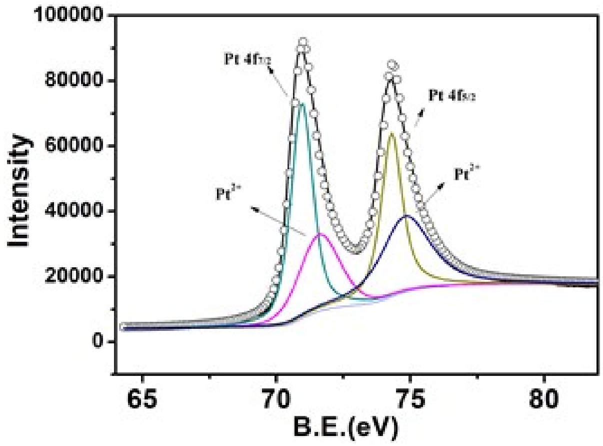

Fig. S1 The XPS results of Pt in Au@PtRu nanorods.

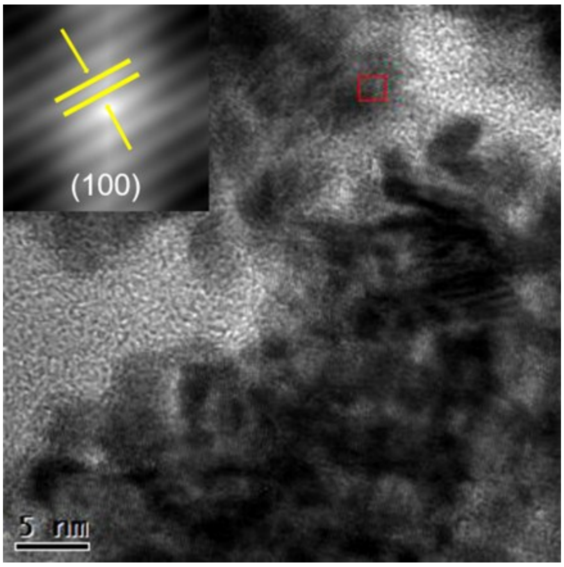

Fig. S2 High-resolution transmission electron microscopy (HRTEM) image of Au@Pt nanorods.

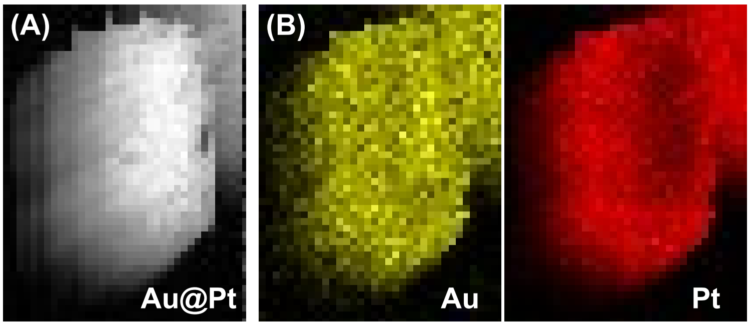

Fig. S3 Scanning TEM (A) and elemental mapping patterns of Au@Pt nanorods (B).

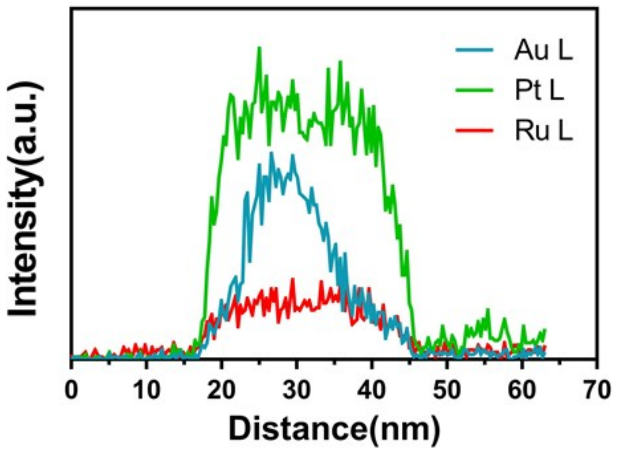

Fig. S4 The compositional line profiles of a single Au@PtRu nanorod.

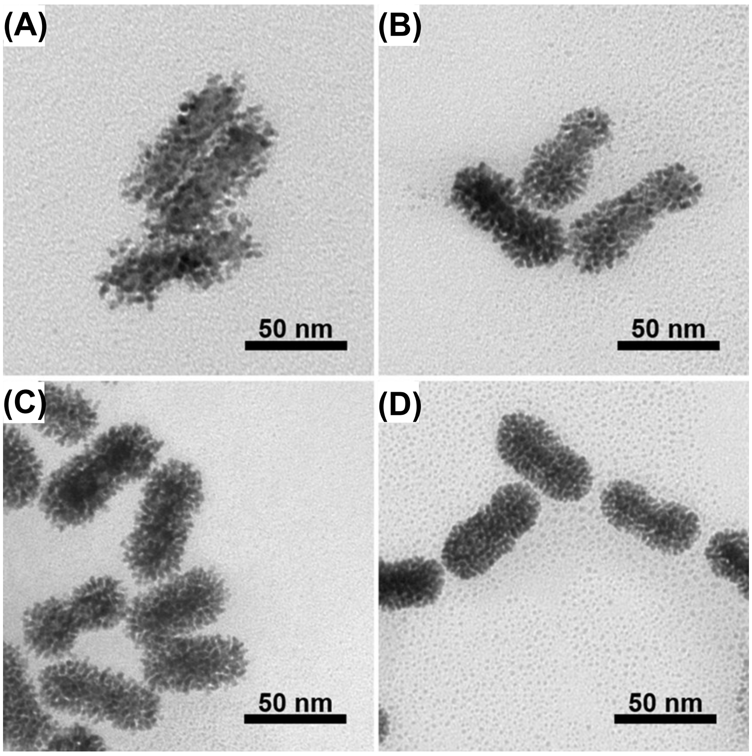

Fig. S5 TEM images of the formation of Au@PtRu nanorods at different time points. (A) 30 min, (B) 60 min, (C) 90 min, (D) 120 min.

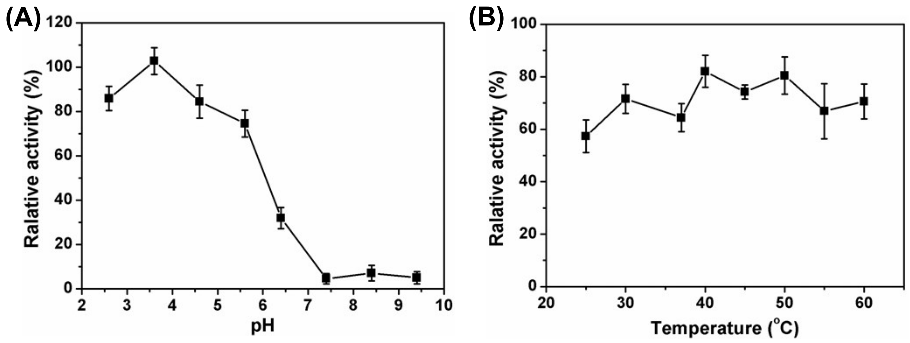

Fig. S6 The effect of pH (A) and temperature (B) on the catalytic activity of Au@PtRu nanorods.

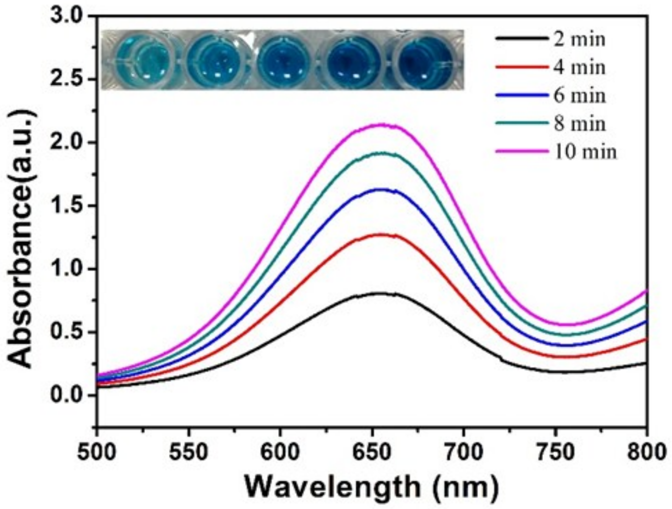

Fig. S7 Time-dependent absorption spectra of TMB catalyzed by Au@PtRu nanorods.

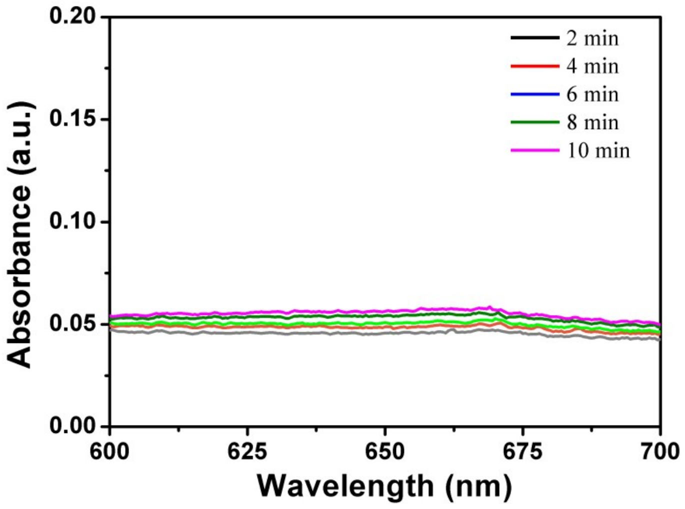

Fig. S8 UV-vis spectra for detection of alcohol oxidase-like activity of Au@PtRu nanorods. The reaction was carried out in 800 μL NaAc-HAc buffer solution (200 mM, pH 3.6), 50 μL ethanol, 50 μL Au@PtRu nanorods (20 μg/mL), and 50 μL TMB (40 mM).

Table S1 Comparison of the kinetic parameters of various catalysts toward the oxidation of TMB by H_2O_2.[a]

Catalyst	Substrate	V_{max} (M s^{-1})	K_m (M)	k_{cat} (s^{-1})	k_{cat}/K_m (s^{-1} M^{-1})	Refs
**HRP**	TMB	1.0×10^{-7}	4.3×10^{-4}	4.0×10^{3}	9.3×10^{6}	S1
	H_2O_2	8.7×10^{-8}	3.7×10^{-3}	3.5×10^{3}	9.5×10^{5}	
**Au@PtRu nanorods**	TMB	1.3×10^{-6}	7.0×10^{-4}	3.4×10^{5}	4.9×10^{8}	Present work
	H_2O_2	1.5×10^{-6}	2.3×10^{-1}	4.0×10^{5}	1.7×10^{6}	
**Fe_3O_4 naoparticles**	TMB	3.4×10^{-8}	9.8×10^{-5}	3.0×10^{4}	3.0×10^{8}	S1
	H_2O_2	9.8×10^{-8}	1.5×10^{-1}	8.6×10^{4}	5.7×10^{5}	
**Graphene oxide nanosheets**	TMB	3.5×10^{-8}	2.4×10^{-5}	2.9×10^{1}	1.2×10^{6}	S2
	H_2O_2	3.9×10^{-8}	4.0×10^{-3}	3.3×10^{1}	8.2×10^{3}	
**Pt nanoparticles**	TMB	1.3×10^{-6}	1.2×10^{-4}	2.3×10^{4}	1.9×10^{8}	S3
	H_2O_2	1.9×10^{-6}	7.7×10^{-1}	1.6×10^{4}	2.1×10^{4}	
**Pd nanocubes**	TMB	9.7×10^{-8}	5.4×10^{-5}	6.9×10^{4}	1.2×10^{9}	S4
	H_2O_2	6.5×10^{-8}	7.0×10^{-1}	4.6×10^{4}	6.6×10^{4}	
**Ru nanoparticles**	TMB	1.3×10^{-7}	6.0×10^{-5}	1.3×10^{4}	2.2×10^{8}	S5
	H_2O_2	7.4×10^{-8}	3.2×10^{-1}	7.0×10^{3}	2.2×10^{4}	
**Fe_2O_3 nanoplates**	TMB	3.9×10^{-7}	5.8×10^{-4}	2.1×10^{4}	3.6×10^{7}	S6
	H_2O_2	3.9×10^{-6}	4.5×10^{-1}	2.1×10^{5}	4.7×10^{5}	
**Au/Fe_3O_4 nanocubes**	TMB	5.9×10^{-7}	4.3×10^{-5}	7.1×10^{2}	1.7×10^{7}	S7
	H_2O_2	4.7×10^{-7}	1.4×10^{-1}	5.7×10^{2}	4.1×10^{3}	

[a)]K_m is the Michaelis constant, V_{max} is the maximal reaction velocity, k_{cat} is the catalytic constant that equals V_{max}/[E], and k_{cat}/K_m is the catalytic efficiency.

References:

1. L. Gao, J. Zhuang, L. Nie, J. Zhang, Y. Zhang, N. Gu, T. Wang, J. Feng, D. Yang, S. Perrett and X. Yan, *Nat Nanotechnol*, 2007, **2**, 577-583.
2. Y. Song, K. Qu, C. Zhao, J. Ren and X. Qu, *Adv Mater*, 2010, **22**, 2206-2210.
3. Z. Gao, M. Xu, L. Hou, G. Chen and D. Tang, *Anal Chim Acta*, 2013, **776**, 79-86.
4. X. Xia, J. Zhang, N. Lu, M. J. Kim, K. Ghale, Y. Xu, E. McKenzie, J. Liu and H. Ye, *ACS Nano*, 2015, **9**, 9994-10004.
5. H. Ye, J. Mohar, Q. Wang, M. Catalano, M. J. Kim and X. Xia, *Sci Bull*, 2016, **61**, 1739-1745.
6. M. Zhu, Y. Dai, Y. Wu, K. Liu, X. Qi and Y. Sun, *Nanotechnology*, 2018, **29**, 465704.
7. M. K. Masud, S. Yadav, M. N. Islam, N. T. Nguyen, C. Salomon, R. Kline, H. R. Alamri, Z. A. Alothman, Y. Yamauchi, M. S. A. Hossain and M. J. A. Shiddiky, *Anal Chem*, 2017, **89**, 11005-11013.
